# T216. THE CULTURAL EXPERIENCES OF PATIENTS DIAGNOSED WITH SCHIZOPHRENIA IN SOUTH AFRICA

**DOI:** 10.1093/schbul/sby016.492

**Published:** 2018-04-01

**Authors:** Wilna Basson

**Affiliations:** Sefako Makgatho Health Sciences University

## Abstract

**Background:**

Schizophrenia is a debilitating mental illness that affects people from all walks of life. Individuals attach meaning to their illness based on their cultural point of view; for some traditional black South Africans, causes of ill health are ascribed to culturally laden inferences. Some patients seek spiritual help before consulting medical doctors. This study aimed to explore how black South Africans diagnosed with schizophrenia experience their illness from their cultural point of view.

**Methods:**

The study followed a hermeneutic phenomenological approach. In-depth interviews were conducted with three patients diagnosed with schizophrenia and on medication for their illness. Their stories were analysed using thematic content analysis.

**Results:**

Five themes emerged during the study. Theme 1 related to Naming Things. The name given to their illness significantly affected the meanings that were attached to the illness. Theme 2 referred to Being Without. Losses as well as gains became apparent. Participants had lost their roles, independence and intimacy; however, they developed other coping strategies and some relationships became stronger. Theme 3 pertained to Connections and Disconnections. While participants were connected to their families and their community, they also felt disconnected due to the stigma perceived. Theme 4 was the theme of Being Spiritual. Spirituality played a vital role in how participants attached meaning to their illness, and it helped them to cope with challenges. Theme 5 was Rainbow after the Rain. The negative connotation of having a mental illness turned into personal, inter-personal and spiritual growth. The devastating illness became a gift to all participants; they demonstrated immense levels of resilience and they found their own way of being and relating.

**Discussion:**

Culture played a crucial role during the initial stages of the illness; all participants sought spiritual help and it determined the meanings attached to the illness. This study proposes a need for mental health workers to explore the challenges that hamper openness within families and communities in order to lessen the perceived stigma experienced by the patients, and to acknowledge and encourage different coping and meaning-making structures, such as spirituality.

